# Digital Primary Care Services, Procedural Justice and Intersectionality: A Critical Realist Approach

**DOI:** 10.1111/1467-9566.70083

**Published:** 2025-09-22

**Authors:** Gina Netto, Farjana Islam, Sara Bailey

**Affiliations:** ^1^ Institute of Place, Environment and Society Heriot‐Watt University Edinburgh UK; ^2^ Institute of Educational Technology The Open University Milton Keynes UK

## Abstract

Healthcare systems in many international contexts have been rapidly digitalised in recent years. Yet, despite the significant transformation of such systems and well‐documented evidence of ethnic inequalities in the use of health services, the consequences of such changes on fairness in decision‐making processes or procedural justice for racially minoritised people have been under‐explored. Further, little attention has been paid to the influence of social determinants of health and systems of oppression, such as racism and patriarchy, on digital healthcare and the ways in which these intersect with each other. We developed a novel critical realist intersectional theoretical and analytical framework to interrogate procedural justice in digital primary care as experienced by racially minoritised people, employing the widely used criteria of voice, trust and impartiality. Analysis of interviews with 100 people from racially minoritised communities in the United Kingdom revealed serious shortcomings with respect to all three criteria, which need to be urgently addressed. We propose a multi‐pronged approach, which recognises high levels of digital poverty, variations in digital literacy and proficiency in English and the need for more attention to the design of digital services and workforce training, along with proactive use of digital services to address existing ethnic inequalities.

## 1 Introduction

Healthcare services in many international contexts have been rapidly digitalised (Matlin et al. 2024; Ho et al. 2024). Yet, despite the far‐reaching consequences of these changes for patients and extensive evidence of the need to address racialised power imbalances that contribute to unequal health outcomes (Ganguli‐Mitra et al. 2022; Nazroo et al. 2020; Powers and Faden 2019), the impact of these changes on procedural justice has yet to be investigated. Procedural justice emphasises fairness in processes relative to outcomes in decision‐making processes (Lind and Tyler 1988) and has been viewed as important by patients (Fondacaro et al. 2005; Murphy‐Berman et al. 1999). Voice, trust in the key actors involved, and the impartiality of the process are widely used as criteria for evaluating procedural justice (Fondacaro et al. 2005; Blasko and Taxman 2018; Manchak et al. 2014). In this article, we incorporate these criteria to develop a novel theoretical and analytical framework for evaluating the procedural justice of digital primary care, a key development in the planning and delivery of healthcare in the United Kingdom, as experienced by racially minoritised people.

By racially minoritised people, we refer to individuals who self‐identify as African, Bangladeshi, Caribbean, Chinese, Indian, Pakistani and mixed ethnic groups. We have focused on these groups because they have been established in the country for several decades (Becares 2015), yet, by virtue of skin colour and other physical features, are vulnerable to processes of racialisation in the institutions within which they participate (Miles 1989). We use the term racially minoritised people interchangeably with minoritised ethnic communities (Bramley et al. 2022), minority ethnic communities (Greenhalgh et al. 2007) and ethnic minorities (Magadi and Magadi 2022), reflecting the terms used to refer to these groups of people in previous research, in the awareness of the considerable diversity that exists between and among the people who self‐identify with these groups.

Our novel theoretical framework integrates intersectionality theory (Collins and Bilge 2016; Crenshaw 1991) within the philosophical stance of critical realism (Bhaskar 1989; Sayer 2000). Along with Krieger et al. (1997), we argue that greater attention to the intersection of the systems of oppression that individuals experience as they attempt to navigate increasingly complex healthcare systems will reveal the multiple social locations and injustices that racially minoritised individuals *simultaneously* experience. Intersectionality theory, first introduced by Crenshaw (1991), is widely used to illuminate how various forms of inequality and discrimination intersect and overlap with each other. The novel critical realist approach to intersectionality theory that we propose enables the identification of ‘constraints’ and ‘enablements’ (Sayer 2000) that interact with multiple dimensions of identity and inequality to respectively hinder and facilitate access to and use of health services.

Our original application of a critical realist approach to intersectionality theory enables us to progress understanding of how health justice can be progressed in four ways. Firstly, it reveals not only the impact of organisational structures, systems and processes on racially minoritised people, but also the role of unequal power relations within the home in individuals' interaction with digital primary care. Secondly, our approach enables us to reveal the influence of both patriarchy and racism in general practices, as well as the impact of patriarchy within the home on racially minoritised women, an area that has been overlooked, and impacts on the procedural justice of digital healthcare. Thirdly, in alignment with the action proposed by the World Health Organization (2008) for progressing health equity, we consider the differential impacts of key social determinants of health in accessing such care, for example, individuals' positions in the housing and labour markets and income, as well as how these locations intersect with language use and migration trajectories. Fourthly, our theoretical innovation not only demonstrates the formidable struggles faced by racially minoritised people in accessing digital primary care but also helps to explain why, under certain conditions, some individuals are able to access such care. This enables us to move beyond ‘static representations of the dominance of oppression to explicitly include the interplay of advantage and disadvantage’ (Rodriguez et al. 2016) in either promoting or hindering procedural justice.

Along with this novel theoretical contribution, we build on the initial analysis of racially minoritised people's engagement with digital primary healthcare (Islam et al. 2024) and Magadi and Magadi’s (2022) analysis of the lower satisfaction levels of ethnic minorities with their general practitioner (local NHS doctors) to increase empirical understanding of the nature and type of injustices experienced in engaging with digital primary care. More positively, our analysis also reveals some grounds for cautious optimism in the form of isolated facilitators or ‘enablements’ (Sayer 2000), which, if proactively built upon and sustained, can begin to counter current injustices perpetuated through such systems.

Having introduced our theoretical and empirical contribution to increasing understanding of procedural justice in the use of digital primary care, in the remainder of this paper we set out the policy context for its implementation in the United Kingdom. We then outline our critical realist approach to intersectionality theory and its utility in assessing the fairness of such care as experienced by racially minoritised people. We next discuss key criteria widely used to evaluate procedural justice, which we apply to data analysis, followed by the research design and methods employed for our empirical research. This is followed by the results of our appraisal of procedural justice and, finally, a discussion in which we highlight the value of our theoretical approach and empirical findings, the methodology employed and its implications for promoting health justice.

### 1.1 Digitalisation of Primary Care Services and Social Determinants of Health

In this section, we identify macro‐level structural factors which impact on the procedural justice of digital primary care as experienced by racially minoritised people through reviewing the policy context in the United Kingdom. Primary care services involve contact with general practitioners (GPs or local doctors) who play a key role in the early diagnosis of illness, treatment and referral of patients to specialist services (Sripa et al. 2019). The transformation of these services through digitalisation had been anticipated as part of the National Health Service (NHS) Long Term Plan 2019a, which states that:

Over the next five years, every patient will be able to access a GP digitally, and where appropriate, opt for a ‘virtual’ outpatient appointment.

In Scotland, where health services have been devolved, a similar emphasis has been placed on the use of digital services in the *NHS Digital Health and Care Strategy* published in October 2021 (Scottish Government 2021). Despite far‐reaching changes in service delivery and the importance placed by policymakers on equity in healthcare access and outcomes (Wadhawan et al. 2023; National Health Service 2019b), the impact of processes of digitalisation on racially minoritised people has received little attention.

In the United Kingdom, the lower satisfaction levels of ethnic minorities with GP services have been extensively documented, with social deprivation and communication difficulties being consistently identified as contributory factors (Magadi and Magadi 2022; Greenhalgh et al. 2007). This is perhaps not surprising in light of previous research which has established high levels of poverty in some of these communities, particularly among Pakistanis and Bangladeshis (Karlsen and Nelson 2021; Marmot et al. 2010, 2020), which are likely to impact on access to digital healthcare, for example, lack of ownership of an adequate smartphone. Further, little attention has been paid to varying levels of proficiency in English, the dominant language used in the health service, among and within ethnic groups, and how this might impact on communication.

Compounding these shortcomings, intersectional analysis of the ways in which racially minoritised individuals' membership of more than one social category impacts on satisfaction levels is lacking, limiting understanding of how ethnicity, gender, age and migration status intersect with each other ‘to either mitigate or magnify the risk of injustice’ (Powers and Faden 2019) within institutionalised systems. The role of social determinants of health in racially minoritised people's engagement with primary care services has also been under‐explored, a gap which we help fill through interrogating the interaction between macro‐level policies and their implementation at the meso‐level of general practices and households, as well as through the micro‐level actions of racially minoritised people, as explained below.

### 1.2 Intersectionality, Critical Realism and Digital Primary Care

We used the critical realist approach to intersectionality theory in this research because of the importance it places on embedding subjectivities within systemic dimensions of power in institutions to reveal their intersections for analysis (Netto et al. 2020; Rodriguez et al. 2016). This approach enables us to expose the patterns of inequality that stem from the racialised and gendered power dynamics operating through general practices (local health centres) in England and Scotland, as well as within the private setting of the home, and to identify the impacts on the procedural justice of digital primary care.

Our approach to intersectionality theory is consistent with the emphasis in critical realism on the causality of structural factors, *regardless of the levels of awareness of individuals of their existence* (Bhaskar 1989). Crucially, structure and agency are viewed as distinct because conflation of both deprives each of the causal efficacy of the other. Conflation of structure and agency may risk voluntarism, where the agency of racially minoritised people in engaging with digitalised primary services is emphasised while overlooking the constraints imposed by macro‐level structural factors, such as the dominant use of English in the health system. Alternatively, conflating structure and agency may risk determinism where structural factors, for instance, the lack of adequately trained staff working within general practices, are emphasised at the expense of agentic factors, such as the ability of some individuals, in certain circumstances, to challenge discriminatory action.

In focusing our attention on the causality of structural factors, we highlight the contingency of single contextual factors operating at the meso‐level of households, such as limited access to the internet or adequate digital devices, on other contextual factors, such as gendered power dynamics operating in general practices or in the home, in bringing about certain effects (Sayer 2000). We demonstrate that revealing the interaction of factors operating in the home as well as general practices more comprehensively illustrates injustices in the operation of digital primary care than simply focusing on either the general practice or the home. Further, we recognise the role of important social determinants of health, particularly housing, low income and employment, which impact on engagement with digital primary care. Significantly, we also demonstrate how, in some contexts, individuals succeed in engaging with these services due to the interaction of certain structural factors at the meso‐level of the general practice, such as the presence of bilingual staff, with their agency and personal characteristics. Examples of personal characteristics that facilitate engagement with digital primary care include highly developed digital literacy or social support characteristics, such as access to relatives who are able to help them.

Using the language of critical realism, for instance, Sayer (2000), we argue that clusters of conditions—both ‘constraints’ or barriers and ‘enablements’ or catalysts—at the meso‐level of the home, as well as general practices, hinder or facilitate access to and engagement with digital services. An example of a cluster of constraints in the home for racially minoritised people is lack of adequate digital devices, absence of informal support and limited proficiency in English. In general practices, an example of such a cluster may include the use of English as the only language for communication and the racist attitudes or stereotypical assumptions of some health professionals. Conversely, clusters of enablements in the home may include individuals' access to informal support, adequate digital devices and high levels of proficiency in English. In general practices, clusters of enablements may include user‐friendly digital platforms and well‐trained staff. By revealing the complex interplay of barriers and facilitators in engaging with digital primary care in the home as well as general practices, our theoretical innovation enables us to more fully explore the implications for developing policy and organisational changes for promoting procedural justice than had we focused solely on the operation of general practices or the power dynamics and conditions in individuals' households.

In the next section, we discuss the incorporation of widely used criteria in appraising procedural justice in our framework.

### 1.3 A Critical Realist Intersectional Approach to Appraising Procedural Justice

Procedural justice has been viewed as synonymous with *procedural fairness* (Adler and Henman 2001), which is concerned with the fairness with which decisions are made within healthcare organisations, particularly among patients, and independent of outcome considerations (Murphy‐Berman et al. 1999). Using three widely applied evaluative criteria, Fondacaro et al. (2005) found that overall, procedural justice dimensions were significantly related to patient satisfaction. These criteria were voice or participation, trust and impartiality. These findings have been supported by other studies which have predicted patient satisfaction. For instance, Tucker et al. (2013) found that predictive relationships of patient satisfaction were mediated by trust in their provider and perception of provider impartiality. Below, we consider the three criteria further, including how constraints and enablements may operate at the macro‐, meso‐ and micro‐levels in ways which impact the procedural justice of digital primary care as experienced by racially minoritised people in the United Kingdom.

Voice has been identified as encompassing both process control (control over input into the decision‐making process) and decision control (control over the actual decision made) (Thibault and Walker 1975). The criterion may be viewed as patients' opportunity to freely communicate and discuss health concerns with health professionals and to participate in decision‐making on treatment options. Our framework enables us to identify how factors operating at the macro‐level of the NHS, such as the dominant use of English across the system, present formidable constraints to individuals at the meso‐level of general practices.

Trust was identified by Fondacaro et al. (2005) as being associated with patients' feelings of comfort about the way their provider handled the situation and the extent to which they provided them with as much information as possible about their medical condition and options for treatment. Trust operates temporally in that its gradual accumulation or erosion over time could mediate relationships between other procedural justice dimensions, such as voice and impartiality. Our critical realist approach to evaluating trust enables us to identify constraints and facilitators at the meso‐level of general practice which influence individuals' confidence that health professionals are acting in their best interest.

Impartiality is associated with whether individuals feel they are being treated in a respectful and nondiscriminatory manner, without bias and with concern for them as individuals (Fondacaro et al. 2005). Use of this criterion compels recognition of the operation of institutional racism as well as interpersonal micro‐level racisms occurring within general practices. Recognising the links between institutional and interpersonal racism allows us to reveal how interpersonal racism is supported and sustained through implementing digital primary care as well as how this process undermines the procedural justice of such care. Having set out and developed key components of our theoretical and analytical framework, we now describe the methods for the empirical research that this paper draws on.

## 2 Methods

To investigate the macro‐level structural factors which impacted on the NHS at the national level in the United Kingdom and which were implemented at the devolved level of Scotland, we reviewed key policy documents which indicated government encouragement and support for transition to digital healthcare and the timescales within which these were expected. These structural factors set the context within which we investigated the procedural justice of digital primary care at the meso‐level of general practices and households, as well as the micro‐level interactions of individuals with health practitioners at the local level.

To investigate the constraints and facilitators in general practices and households, we employed qualitative research methods in the form of in‐depth narrative interviews in England and Scotland. Fieldwork was carried out between August and December 2022. We worked in partnership with four community organisations to recruit 100 women and men from African, Bangladeshi, Caribbean, Chinese, Indian, Pakistani and mixed ethnic groups in four urban areas, chosen because of the high representation of these ethnic groups in the local population: the London Borough of Tower Hamlets and the cities of Manchester, Bradford and Glasgow. We adhered to the highest level of ethical standards in the recruitment, conduct and analysis of interview data. Ethical approval was obtained from the research ethics committees of the institutions within which the researchers were based (Heriot‐Watt and The Open University). It was also obtained from the National Research Centre on Privacy, Harm Reduction and Adversarial Influence Online as a condition of grant funding.

We employed a purposive sampling strategy which enabled us to pay focused attention to the ethnic groups most at risk of digital exclusion due to their disadvantaged socioeconomic status, namely, the Bangladeshi and Pakistani communities (Marmot et al. 2020), as well as other racially minoritised people. We also purposively sampled women and men from three age groups: young (18–35), middle‐aged (36–64) and older (65 and above). The majority were women (60%). Participants were informed of the aims of the research, that information provided would be treated as confidential and that they could withdraw from the research at any point.

Most of the interviews were undertaken in English, with 14 interviews conducted in other languages, either by a bilingual researcher or with interpreting support. On average, interviews took an hour and were undertaken either in person or online via Zoom, depending on participants' preferences and ability to engage with virtual meeting platforms. Almost all the interviews were audio‐recorded and professionally transcribed. A small number of participants consented to being interviewed but not recorded; in these cases, handwritten notes were made. All collected information was anonymised, coded in NVivo and thematically analysed.

### 2.1 Data Analysis

The lead author conceptualised the development of a novel critical realist intersectional lens that incorporated procedural justice criteria to interpret the data generated from the interviews and identified initial codes that led to the construction of themes (e.g., access to digital services and barriers to services). The second and third authors independently coded the data and generated sub‐themes (e.g., digital connectivity, intergenerational support) which revealed the rootedness of participants' engagement with digital primary care and social determinants of health, particularly employment, income and housing. The development of sub‐themes enabled all three authors to identify ‘constraints’ and ‘enablements’ which operated singly or interacted with each other.

All three authors examined participants' engagement with digital primary care against the three criteria of voice, trust and impartiality in appraising procedural justice. Part of this analysis included the development of a small number of instrumental case studies (Stake 1995), which contextualised individuals' engagement with digital healthcare within their migration histories and explored the intersection of age, gender and language use. Such biographical methods have been recommended for deepening understanding of the role of structural features in conditioning actions and meaning‐making processes (Halfacree and Boyle 1993), in this case, in illuminating the impact of social determinants of health on procedural justice in digital primary care.

## 3 Results

Below we discuss the results of data analysis against the three criteria identified by Fondacaro et al. (2005) for assessing patients' appraisals of justice in healthcare decision‐making, that is, voice, trust and impartiality, focusing particularly on voice. Voice emerged as the criterion that was most prominent in the dataset generated by our research and is therefore considered more fully than trust and impartiality below. Issues related to the latter two criteria are considered together because many of the issues overlap with each other.

### 3.1 Voice

Although both voice as process control and over the actual decision made provide the opportunity to be heard (Fondacaro 1995), process control constraints emerged particularly strongly. Below, we discuss the multiple ways in which process control is undermined, including through lack of choice in the availability of non‐digital options and the language of communication, digital poverty and housing conditions or homelessness.

Feelings of frustration and loss were strongly expressed by English‐speaking individuals who felt forced to use digital technology instead of communicating verbally to discuss their health:

Everyone, everywhere, is trying to just get you online … We don't want to speak to you … We don't want to hear the upset or the disappointment. Just type it in and we'll get back to you. For me, that doesn't work that well.

(Black Caribbean female, 50)

They always tell me, ‘Why do you walk in here? There's an app, why don't you do that?’ I say, ‘For me, when I am there, I can’t express my urgency.’ I can’t say my reason really because most of the things are drop down choose … they give you the option of three or four and none of the options is what you want to say.

(Black African male, 53)

As indicated in both quotations, in some general practices, booking an appointment required the patients to either fill in details of the symptoms that they were experiencing or choose from a drop‐down list of symptoms. A common problem encountered here was lack of knowledge of appropriate terminology, such as how to refer to different parts of the body, and the inability to identify symptoms from the available options.

The psychological impacts of reduced opportunities to communicate in person with a GP and input into decision‐making about one's own health may be more generally shared with the wider population. This is likely particularly among individuals who experience digital exclusion, highlighted as a major problem in a recent UK House of Lords (2023) report. Barriers to digital inclusion included the lack of affordable internet access, shortcomings in internet connectivity and inaccessible digital services. These obstacles are likely to be compounded among those whose proficiency in English is limited and could contribute to delayed diagnosis and access to appropriate treatment due to a lack of effective communication with health professionals.

Analysis of narratives revealed overwhelming evidence that the injustices perpetuated through the linguistic insensitivity of health services prior to digitalisation (Greenhalgh et al. 2007; Piacentini et al. 2019) have persisted. At the macro‐level, the standard use of English in the health service, and the shift, in many cases, to written communication, for instance, to book an appointment, is likely to differentially impact racially minoritised peoples' ability to initiate or have some influence in health‐related decisions in interactions with clinicians. As might be expected, individuals who are in the early stages of developing proficiency in English emerged as facing particularly formidable challenges in voicing their concerns.

Indicating ethnic diversity in English language proficiency, after White British people, individuals categorised as ‘Black Caribbean’ were the most likely to speak English as their main language (Office for National Statistics 2013). In contrast, individuals of Bangladeshi origin were the least likely to speak English well. Reinforcing the value of an intersectional perspective that considers the intersection of ethnicity with gender, women of Pakistani and Bangladeshi origin were five times more likely than their male counterparts to speak no English at all, indicating the role of racialised as well as gendered patterns in engaging with digital healthcare.

Although statistical analysis by age was not available, data analysis revealed that most older people, particularly first‐generation migrants, had limited proficiency in English and could not interact with digital platforms. Feeling compelled to use a language in which one is not proficient to communicate symptoms in digital interactions with health practitioners is likely to present significant constraints to voice. The case of a Chinese male who had migrated from Hong Kong in the 1980s and who could only find work in a Chinese restaurant illuminates the significant and persistent role of racialised and gendered patterns of migration and employment in developing proficiency in the language. As he recalls, limited exposure to English and ability to write in the language presented major challenges to communicating digitally:

[Describing my symptoms in English] would be difficult for me … it's difficult because the English is difficult to type in all that … the struggle is how to describe the pain in their standard.

(Chinese male, 70)

These findings highlight the need for greater attention to supporting the shift to written communication. The standard use of English in ‘routine processes and procedures’ (Nazroo et al. 2020) may be viewed as a manifestation of macro‐ and meso‐level institutional racism, which undermines procedural justice through a lack of attention to empowering racialised minorities to participate in decision‐making processes.

In one of the few interviews that evidenced linguistic sensitivity within a general practice, clusters of language‐related enablements were revealed by a Black African man, aged 82, who spoke about the experience of someone he knew who had limited English proficiency. The individual concerned was supported through interpreters working with receptionists and GPs at two stages: booking an appointment online and during an online consultation. However, this demonstration of linguistic sensitivity emerged as very much the exception rather than the norm.

Women who were not economically active, in low‐income households and who had limited digital literacy emerged as particularly disadvantaged. For instance, the case of Mrs H, a middle‐aged Bangladeshi woman who had joined her family in the United Kingdom a few years ago and who spoke limited English, reinforces how recent arrival in the country, patriarchy, domestic tensions, lack of financial independence, limited digital literacy and educational opportunities interact with each other to seriously hinder decision‐making in digital healthcare:

Our (conjugal) friction is cost and Internet bills … My husband does not pay for my mobile internet … I am a jobless woman … I don't know much about IT classes here … Who will tell me?

(Bangladeshi female, 54)

In contrast, highlighting the value of a rigorous intersectional analysis which examines how ethnicity intersects with gender and age in process control, young and middle‐aged women who were digitally literate and proficient in English emerged as not only confident in using digital primary care but also actively assisting older people to do so, though not without some costs to themselves:

My parents, who are older, they wouldn't know how to take a picture … send a picture to a doctor.

(Asian‐British female, 34)

I've actually downloaded it (GP Apps) for my mum and dad … so that I can order their prescriptions.

(British Bangladeshi female, 37)

I do help my parents out as well sometimes, and it just gets a bit [pause] … and the kids accounts as well, so there are a lot of accounts.

(Pakistani female, 38)

The extracts above reveal some of the tasks that informal carers help with, such as taking and uploading photographs for diagnostic purposes, booking appointments, downloading health applications and ordering prescriptions. They also reveal the often time‐consuming nature of this care, with consequences for their personal health and well‐being. Consequently, the unpaid contribution of informal carers, disproportionately women, in supporting older people to input into decisions about their health in digital primary care may be read as constituting a gendered element of procedural injustice for the carers involved.

Reinforcing the close relationship between marginalisation in the labour market and voice, digital poverty featured as a recurrent barrier and a manifestation of the wider poverty experienced by individuals from some groups (Marmot et al. 2010, 2020). We found that at the micro‐level, digital primary care was often hindered through lack of access to suitable devices:

I use my smartphone for now, but I always used to have a computer … but that's not working now.

(Black African female, 44)

My phone quality is really bad … For my skin conditions I had to send a picture. They called back saying the quality is not good … So I tried to do it with my husband's phone – thank God for that … and they prescribed a medication.

(Bangladeshi female, 42)

These extracts reveal the contingency of accessing digital healthcare on the affordability of digital devices, as well as gendered inequalities in the quality and ownership of these resources, revealing the interaction of three meso‐level constraints at the household level among some racially minoritised people.

Highlighting the relevance of housing as a social determinant of health, homelessness or staying in insecure housing is also likely to disrupt process control in digital healthcare:

I did have the broadband, but I'm in the process of moving … so I'm not renewing it till I move … I don't have any internet now.

(Bangladeshi female, 38)

Oh, I've been on the council waiting list for such a long time. I became homeless, and from homeless, after maybe 20 years later, I have a permanent house now.

(Bangladeshi female, aged 52)

Here it is worth noting that Black and minoritised ethnic communities in the United Kingdom, taken as a whole, experience higher levels of homelessness (Bramley et al. 2022), impacting on internet coverage. Although statistical analysis of the way in which gender intersects with ethnicity to impact homelessness is not available, some of the housing insecurities which women experienced emerged in our research, revealing the precarity of their engagement with digital healthcare. For instance, Mrs B, an older Bangladeshi widow, expressed considerable distress and fear that she might lose her council home due to her husband's death and her immigration status as an individual with ‘no recourse to public funds’. Her case clearly highlights the contingency of digital connectivity on living in secure accommodation, as well as how gender can intersect with age, immigration and marital status to hinder voice in healthcare.

Having considered the complex ways in which gender and age intersect with ethnicity, migration trajectories and the social determinants of health to undermine opportunities to input into decision‐making about healthcare, we now appraise the procedural justice of digital healthcare among racially minoritised people using the criteria of trust and impartiality.

### 3.2 Trust and Impartiality

Interview analysis revealed that participants' willingness to trust in digital primary care was related to multiple factors, including uncertainty about the responsiveness of local doctors:

The worst thing is, even once you fill up the consultation form, you don't know if the doctor's going to call you. There's no acknowledgement to say, ‘The doctor is going to call you’.

(Black African male, 48)

You fill it, you wait, no‐one replies to you, so for me online is good for other people, but for me, I prefer the phone. So I speak with now, and when I speak, I ask your name. Okay, I know that I speak with this person, yes.

(Black African female, 47)

The quotes above reflect a lack of trust in digital healthcare to facilitate timely contact with a doctor to discuss health‐related concerns. In the second quote, the need to liaise with an identifiable individual about healthcare needs was also emphasised, highlighting the need for personal contact to inspire trust. The distrust expressed by these patients may appear similar to that experienced by others in the wider population. However, noting that trust operates temporally, such feelings may be compounded by a lack of opportunity to build up rapport over time with local doctors due to digital poverty, limited digital literacy or proficiency in English, or the intersection of all of these constraints as discussed above, as well as experiences of racism and racist stereotyping, which we discuss below in relation to the criterion of impartiality. As Fondacaro et al. (2005) point out, distrust could combine with lack of voice in contributing to decision‐making and amplify procedural injustices.

Yet others expressed feelings of anxiety and fear around the use of digital services:

Using digital services makes me feel anxious and scared. That's why I prefer a simple phone call.

(Pakistani female, 50)

I'm concerned about being surveilled, tracked. I mean it's happening already, I guess, but I don't want to contribute to it further. The least I contribute, in my mind, the better for me.

(Black Caribbean female, 50)

The first quote above reflects a lack of trust in the impersonal nature of digital healthcare in contrast to the more personal human interaction possible through a phone call. As indicated in the last quote, some of the distrust could be traced to apprehension that the information supplied could be misused, which, as in the case of the individual quoted directly above, could lead them to withhold potentially relevant personal health information or, worse, disengage completely from digital systems.

Lack of trust was also expressed in relation to difficulties experienced in engaging with digital healthcare. For example, one research participant, a 57‐year‐old Black African male, challenged the system's trustworthiness by rejecting the claim by health practitioners that it is easy to use their digital system:

They do an online service but it's not user friendly … You need to be a rocket scientist to be able to access that. I've tried four times to get it. They say, ‘Oh, it's easy. All you have to do is [pause]’. No, it's not easy. I'm computer‐savvy. I know what I'm talking about.

Experiences of being stereotyped or treated unfairly prior to processes of digitalisation could also result in a lack of confidence in fair treatment through digital healthcare. For example, a 53‐year‐old Black African male who lacked trust in digital healthcare complained that when attending his general practice, White healthcare practitioners either could not or pretended not to be able to understand his accent and that he had missed out on services he needed as a result. He went on to explain that he often felt as though these practitioners were not going to be able or willing to understand his needs even before he had articulated them:

Because once I am approaching reception – you will see somebody really really thinking that he or she is not going to understand what you tell them even before you say it. You see that somebody is already in a way that she or he is likely not to understand … So by the time you speak you'll have seen some expression which shows this person is already struggling before you even say anything.

This quote speaks powerfully to the racialised dynamics of perception and communication within institutional settings. The speaker describes a moment of being pre‐judged, not based on what they say but on how they appear before they even speak.

Interview analysis also revealed how gender can intersect with processes of racialisation among individuals within the same ethnic group to mediate patients' willingness to trust health professionals. These issues often overlap with participants' concerns relating to impartiality as experienced by Mrs J, a middle‐aged Pakistani woman of 52:

There are more male GPs that are from the South Asian community, but I can't speak to them … They understand and speak the language but I feel like they, in particular, disregard me and don't get to the root of the problem … a woman of my age has intimate problems … and I can't tell my problems to men, even if they speak the same language.

Mrs J's case shows the complex interplay between racialised and gendered patterns of employment in the health service and her willingness to disclose ‘intimate problems’ to doctors who are not trusted due to their patriarchal attitudes towards female patients. Her situation highlights the significance of other barriers to communication beyond a shared language among individuals who belong to the same ethnic group (Piacentini et al. 2019), such as lack of attention to getting ‘to the root of the problem’. Analysis of the NHS workforce in June 2022 revealed that 49.9% of hospital and community health services (HCHS) doctors were ethnic minorities (not including White minorities). Of these, 32.0% were Asian (NHS 2023). Although a breakdown according to gender among Asian staff is not available, Mrs J’s distrust in male doctors who speak the same language may be read as a lack of confidence in the reproduction of the ‘public gender regime’ (Walby 1997) in her local general practice. Walby (1997) conceptualised a gender regime as a societal patriarchal structure that restricts women and maintains the domination of men. She distinguished between public gender regimes, which operated within institutional structures, and private gender regimes in domestic spaces. According to her, processes of social transformation have resulted in the transformation of the patriarchal strategy of excluding women in the private or domestic sphere to one of segregating and subordinating women in the public gender regime. Such processes are complex, non‐linear and continually evolving. The use of a critical realist intersectional lens enables us to recognise that processes of social transformation such as that involved in the digitalisation of primary care can potentially contribute to the evolution of new public gender regimes. Within such regimes, ethnicity and gender can interact in complex ways to result in women such as Mrs J being less willing to trust that her concerns are being seriously considered among men who belong to the same ethnic group as herself.

Several participants suggested that there was a lack of impartiality regarding whether or not they and others were allowed to use non‐digital means to access primary healthcare services. For example, a 38‐year‐old Bangladeshi woman remarked that although she was instructed to use her GP's digital app to request a service, a friend of hers had been able to negotiate telephone‐based access to care:

I spoke to some of my friends who were saying their GPs, they are allowing them to sometimes call. One of my friends was saying if she puts her foot down, if she makes a fuss over the phone, then they will let her [make an appointment over the phone]. I thought, do we really need to do all that just to speak to our GP?

The friend who ‘puts her foot down’ demonstrates skills in manoeuvring through institutions which do not readily accommodate their needs (Yosso 2005). This suggests that those with more confidence may fare better in an unequal system. It also raises questions about the effort needed to gain access to non‐digital channels of communication. The participant's rhetorical question—‘*Do we really need to do all that just to speak to our GP?*’—reveals the emotional toll of navigating health systems that do not accommodate individual needs and the burden of self‐advocacy within unfair systems.

Shortcomings related to the impartiality of digital primary care may also be illustrated through the experiences of men who struggled to obtain screening for prostate cancer, a condition which African and Caribbean men are more vulnerable to (Martins et al. 2015). Below, a middle‐aged Black African man recalled the challenges that he faced:

Personally, my dad died from prostate cancer, and I didn't have my prostate cancer test until I was 56. It was a struggle getting that blood test … they just don't want to know because of the embedded racism in the system.

(Black African male, 57)

As the quote above indicates, such omissions may be read as manifestations of institutional racism, extending Nazroo et al.'s (2020) definition beyond the translation of ‘routine processes and procedures into actions that shape the experiences of racialised groups within these institutions’ to the omission of actions that could reduce health inequity, such as enabling access to preventative treatment among individuals from ethnic groups that are recognised as being more vulnerable to certain diseases. Such inaction may be viewed as a meso‐level constraint in general practices. In an encouraging example of the potential for digitalisation to be used as an enablement to help counter such examples of institutional racism, a doctor who was aware of the difficulties that African and Caribbean men faced in being tested for prostate cancer made available an online letter that they could take to their general practice to help them obtain such a test.

Yet another example of racialised treatment that some individuals encountered was the lack of support that they experienced when they struggled to access digital care. For instance, Mrs P, a middle‐aged Pakistani woman who had limited proficiency in English and who had tried to use the phone to book an appointment, in some cases with some success, but not always, recalled:

Sometimes to make an appointment I can phone the GP and there's a receptionist there that can speak Urdu. Sometimes she might help me on a good day but most of the time now they tell me that I can't speak to them in Urdu. I try and ask if there's anyone to help me just make an appointment and they just laugh on the phone. I feel insulted.

(Pakistani female, 52)

Her narrative illustrates the variable nature of language support—available on ‘a good day’—to book an appointment and an important manifestation of procedural injustice. At other times, her inability to use the language in which she can most clearly communicate to gain access to a key service might be viewed as an institutional form of linguistic discrimination (Drożdżowicz and Peled 2024). Taking an intersectional perspective that considers the ways in which processes of racialisation interact with gender, as manifested by the lower levels of proficiency in English among females from some ethnic groups compared to their male counterparts as discussed above, women are more likely to encounter such discriminatory behaviour.

Highlighting the vulnerability of such individuals to being overlooked or marginalised and the close relationship between the criteria of voice, trust and impartiality, the position of Mrs S, a middle‐aged woman of Bangladeshi origin who has limited proficiency in English, is illuminating:

I want to speak to somebody face to face. I don't want to be defrauded … I don't want to be ignored. I feel like it's easy to disregard me because I don’t speak the language.

(Bangladeshi female, 52)

Her experience reinforces our analysis that institutional racism in the form of macro‐level linguistic insensitivity to the impacts of digitalisation on communication can be sustained through racialised micro‐level interactions in general practice settings where health professionals fail to take appropriate action to overcome such barriers.

## 4 Discussion

This article investigated the impact on procedural justice of the far‐reaching ramifications arising from the rapid digitalisation of primary care on racially minoritised people, a process that is significantly transforming health services in many international contexts. The research is agenda‐setting in revealing the multiple ways in which ethnicity intersects with multiple aspects of identity and the social determinants of health to undermine the procedural justice of digital primary care. Unless urgently addressed, these injustices are likely to replicate or exacerbate pre‐existing inequalities in accessing healthcare.

By developing a pioneering critical realist approach to intersectionality theory, we have extended conceptual and empirical understanding of the procedural justice of primary care beyond Fondacaro et al. (2005) in three ways. Firstly, we have revealed how justice can be promoted or hindered through structural factors arising from the intersection of important social determinants of health, such as homelessness and housing conditions, location within and outwith the labour market, and income with migration trajectories and multiple aspects of individuals' identities, including ethnicity, age and gender. Secondly, we have shown how justice can be undermined by the interaction of structural constraints at the macro, meso and micro levels of digital health systems. Thirdly, and more positively, it has enabled us to identify enablements that mitigate the impacts of emerging injustices and opportunities for proactively using digitalisation to address existing ethnic inequalities, such as the greater vulnerability of some ethnic groups to certain diseases.

Empirically, the use of the framework has enabled us to extend quantitative research undertaken by Magadi and Magadi (2022), which has revealed the lower satisfaction levels of racially minoritised people with digital primary care, by revealing the multiple ways through which procedural justice has been undermined with respect to three widely used criteria, that is, voice, trust and impartiality. Our investigation into voice revealed differential access to the multiple resources required for engaging with such care, including material resources (such as digital devices) and social support (such as intergenerational care), as well as significant variation in individual attributes, such as digital literacy and proficiency in the dominant language required for effective engagement with digital healthcare. With respect to trust, we have shown how lack of human contact and confidence in the responsiveness of local doctors, disbelief in claims by health practitioners that digital healthcare systems are easy to use, fear that personal information supplied might be misused, anxiety in using digital systems and previous experiences of racial stereotyping undermine the confidence of racially minoritised people that digital healthcare systems will be used to address individual health concerns. Highlighting the close relationship between trust and impartiality, we have also revealed the complex ways in which racialised dynamics can hinder communication, as well as how patriarchy can interact with ethnicity‐related employment patterns in health services to contribute to feelings of being marginalised and treated unfairly. Our analysis has enabled us to extend Nazroo et al.'s (2020) conceptualisation of institutional racism by demonstrating that it is not only perpetrated through ‘routine processes and procedures into actions that shape the experiences of racialised groups within these institutions’ but also through inaction. A clear example is the lack of attention to ensuring the sensitivity of digital platforms for patients with limited proficiency in the dominant language.

Methodologically, aspects of our research design that were particularly useful in undertaking a rigorous appraisal of the procedural justice of digital healthcare included the inclusion of individuals from seven racially minoritised groups in our sample. This enabled us to identify common processes of racialisation that undermined justice across all groups, while enabling us to ensure that the impacts of socioeconomic disadvantage were captured through our purposive sampling strategy of ensuring that groups that were particularly socioeconomically disadvantaged were adequately represented. Purposive sampling based on gender enabled us to capture patriarchal attitudes that resulted in unequal access to digital resources and opportunities to gain digital literacy within some households, as well as the disproportionate impact on young and middle‐aged women who assisted older relatives as well as children in accessing digital care. Finally, the multi‐sited nature of our study enabled us to capture common injustices arising from digital primary care across the country despite diversity in terms of the ethnic composition of local populations and differences between central and devolved government in introducing digital healthcare. A limitation of the methodology is its focus on digital primary care in the United Kingdom, a shortcoming that needs to be addressed through similar theoretically underpinned approaches that interrogate the procedural justice of digitalising primary care systems in other international contexts.

As digital systems continue to be rolled out in the United Kingdom as well as globally, our research highlights the need for a multi‐pronged approach for digitalising healthcare while recognising the importance of face‐to‐face interaction to facilitate communication in some circumstances. Firstly, at the macro level of government, greater recognition is required of the urgent need to address digital poverty and the ways in which this intersects with ethnicity and gender to compromise active involvement in decision‐making in digital healthcare. Secondly, increased awareness is needed of the potential for digitalisation to result in challenges in accessing timely healthcare, which need to be addressed by easy access to non‐digital options as well as sources of support that can enable racially minoritised people to take effective action. Thirdly, greater awareness is needed among policymakers and designers of digital tools of limitations arising from limited digital literacy and its interaction with limited proficiency in the dominant language for communication, alongside efforts to increase digital skills and proficiency in the language among racially minoritised people. Fourthly, greater attention to building workforce capacity in engaging with an ethnically diverse population within a wider culture of anti‐racist practice is required to ensure adequate support for patients in accessing digital primary care. Fifthly, greater awareness is needed of the potential for digital primary care to be proactively used to develop interventions targeted at sub‐groups of ethnically diverse patients who are vulnerable to certain diseases to ensure more equitable outcomes over time.

We have demonstrated that centring the perspectives of racially minoritised people provides a uniquely helpful vantage point for evaluating the procedural justice of evolving digital systems and indicating the agenda for positive change. This approach has also enabled us to contribute to efforts to advance procedural justice through co‐creating and producing multiple resources, including multilingual videos for racially minoritised people and community organisations, informing the development of a code of practice for designers of digital tools to help produce more ethical, trustworthy and transparent digital tools and free‐to‐use privacy‐enhancing and other technologies which enable easy identification of the concerns of racially minoritised people and assist in the identification of geographical areas which would benefit from additional investment (https://www.primecommunities.online/). However, although we hope that such resources and tools will be helpful, they cannot take the place of high‐level recognition of the need for equitable and sustainable digital health policy and investment.

Finally, although we have focused on the procedural justice of digital primary care in the United Kingdom for racially minoritised people, our novel theoretical and analytical framework and the resources that we have developed can be adapted for use in other institutional and international contexts. The framework can also be adapted to examine the ways in which other systems of oppression, such as ableism and heteronormativity, interact with racism, patriarchy or both to progress or undermine the procedural justice of healthcare.

## Author Contributions

**Gina Netto:** conceptualization (lead), data curation (equal), formal analysis (equal), funding acquisition (lead), investigation (lead), methodology (lead), resources (lead), supervision (lead), validation (lead), writing – original draft (lead), writing – review and editing (lead). **Farjana Islam:** conceptualization (supporting), data curation (equal), formal analysis (supporting), funding acquisition (supporting), investigation (supporting), methodology (supporting), resources (supporting), validation (equal), writing – original draft (supporting), writing – review and editing (supporting). **Sara Bailey:** conceptualization (supporting), data curation (equal), formal analysis (supporting), investigation (supporting), methodology (supporting), resources (supporting), validation (equal), writing – original draft (supporting), writing – review and editing (supporting).

## Consent

The authors have nothing to report.

## Conflicts of Interest

The authors declare no conflicts of interest.

## Permission to Reproduce From Other Materials

The authors have nothing to report.

## Data Availability

The data used for this study are not publicly available for ethical reasons. The qualitative nature of the research generated highly personal and sensitive information from individuals from racially minoritised communities, including information about their ethnicity and migration status. Ethnicity is recognised as a ‘protected characteristic’ under the UK's Equality and Human Rights Act 2010.
